# Spontaneous regression of congenital epulis: a case report and review of the literature

**DOI:** 10.1186/1752-1947-4-331

**Published:** 2010-10-21

**Authors:** Priyanshi Ritwik, Robert B Brannon, Robert J Musselman

**Affiliations:** 1 Department of Pediatric Dentistry, LSUHSC School of Dentistry, New Orleans, USA; 2 Department of Oral and Maxillofacial Pathology, LSUHSC School of Dentistry, New Orleans, USA

## Abstract

**Introduction:**

Congenital epulis is a rare lesion found on the alveolar process of a newborn child, diagnosed soon after birth. The lesion has a site predilection for the anterior maxillary alveolar process and a 9:1 sex predilection for females. Once diagnosed the traditional management of the lesion has been surgical excision under general anesthesia.

**Case presentation:**

The purpose of this case report is to describe spontaneous regression of congenital epulis in a three week old healthy African American female child. She presented with a 1.5 cm bilobed sessile nodular lesion in the region of the right maxillary cuspid. The clinical impression was congenital epulis. Since the lesion was not interfering with feeding and respiration, a conservative approach was taken. The child was followed-up for 18 months, during which the lesion progressively regressed.

**Conclusions:**

Conservative management prevented unnecessary surgery and anesthesia exposure in a neonate.

## Introduction

The congenital granular cell epulis (CE) is a benign tumor arising from the alveolar ridges of newborns and composed of nests of cells with granular cytoplasm set in a prominent vasculature [1]. Neumann is credited in documenting the first case of CE [2]. In 1871 he described a red smooth-surfaced bilobed tumor resembling a polyp that was attached by a stem to the gums on the left jaw's upper edge of a normally built/shaped newborn. He portrayed the tumor as being composed of large coarse-grained cells with numerous blood vessels that was separated from the overlying oral mucosa by a loosely defined boundary. Neumann's search of the literature for similar cases was for naught. To date there have been over 200 cases of CE reported in the English-language literature [3] with surgical removal advocated as the treatment of choice. There is very limited discussion in the literature about a conservative approach to CE. Because of the paucity of cases treated non-surgically, this report describes the clinical features and biologic behavior of a CE that resolved without surgical intervention. This report also compares these findings with those CE previously reported to have undergone spontaneous remission.

## Case presentation

**Figure 1:**
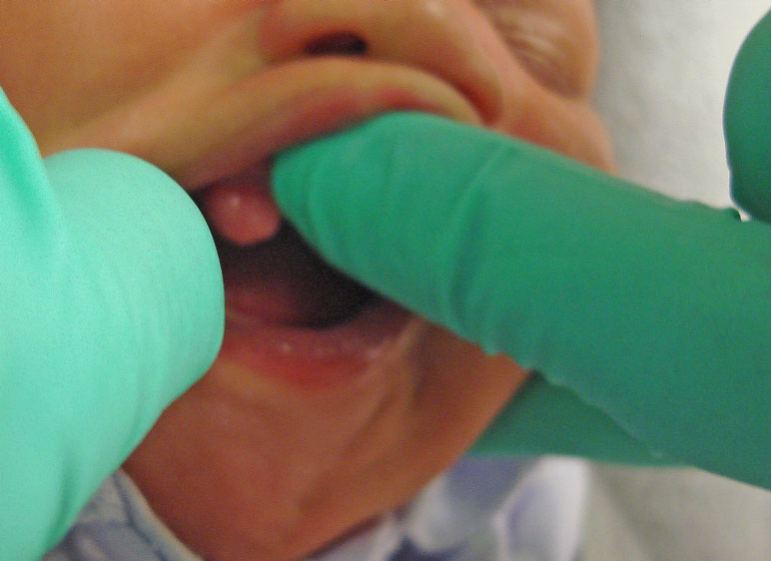
**Lesion at age three weeks**.

**Figure 2:**
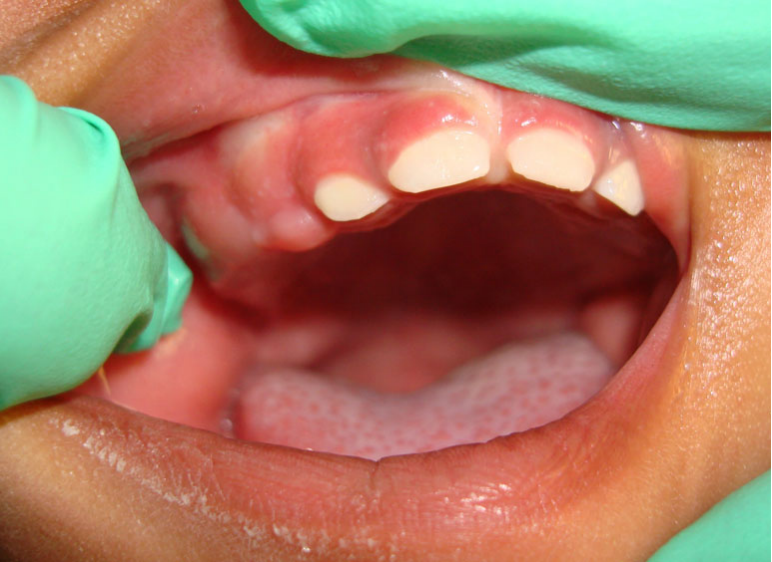
**Lesion at age 16 months**.

A three-week-old African American female child was referred to our clinic for the "evaluation and treatment of cysts in her gums". The child was born at full term via vaginal delivery. She had no other medical problems. Her mother reported that pre-natal history was unremarkable, and that the child was born with a lesion in her mouth. In her mother's opinion the lesion had reduced in size over the three-week duration. Intra-oral examination revealed a bilobed sessile nodular lesion approximately 1.5 cm size in its greatest dimension on the right maxillary alveolar process, in the region of the unerupted canine (Figure 1). The surface of the lesion was smooth, healthy pink and non-hemorrhagic. Upon palpation, there was no pain, discomfort or lymphadenopathy. A maxillary peri-apical radiograph was taken which revealed no radiographic abnormality. The clinical impression was that of CE. Urine analysis to assess vanillylmandelic acid (VMA) to rule out neuroectodermal tumor of infancy was negative. A complete blood count with differential revealed all blood values within the normal range. Her mother preferred non-surgical management of the lesion if it was possible. In consultation with the oral and maxillofacial pathologist it was decided to appoint our patient for weekly observations for a month, followed by monthly observations. Her mother maintained all scheduled appointments. Over 18 months of follow-up of our patient, clinically the lesion reduced to less than 2 mm in size and remained a sessile lesion (Figure 2). The primary dentition is erupting in the maxillary arch without any complications. Our patient has met all developmental milestones for her age.

## Discussion

CE is also known as CE of the newborn [4], congenital granular cell tumor [4], congenital granular cell lesion [4], gingival granular cell tumor of the new born [1] and Neumann's tumor [2]. CE is usually diagnosed at birth; although, if the lesion is large, it may be diagnosed *in utero *by 3D ultrasound and magnetic resonance imaging (MRI) examinations [5,6]. The lesion has a site predilection for the maxillary alveolar process, lateral to the midline in the region of the primary canine and lateral incisor [4]. It has a 9:1 sex predilection for females [4]. Clinically, it presents as a nodular sessile or pedunculated mass with a smooth normal colored surface [4]. Usually, patients present with a single lesion, although there have been case reports of multiple lesions [7,8] and one case report of a patient with involvement of the alveolar ridge as well as the tongue [9]. The lesion may be large enough to make it difficult for the child to feed and/or may cause airway obstruction [8]. Clinical differential diagnoses for CE include hemangioma, fibroma, rhabdomyoma, rhabdomyoscarcoma, lymphangioma, osteogenic and chondrogenic sarcomas, teratoma and granular cell tumor [5,10].

The traditional management of the lesion has been complete surgical excision under either general anesthesia [11] or local anesthesia [12] within hours [8] to days [11,13] after birth. There is one case report of excision of CE using carbon dioxide laser under general anesthesia in a two-day-old infant [13] and another report on the use of erbium, chromium: yttrium-scandium-gallium-garnet (Er, Cr: YSGG) laser to remove a CE lesion [14]. CE is not known to recur after surgical excision even when the removal has been incomplete. The dentition in the region of lesion usually remains unaffected [15,16]. However, there has been a case reported of hypoplastic maxillary primary left incisor, cuspid, and first molar in the region where a 2.5 cm large CE was surgically removed 11 days after birth [17]. Mucoperiostial flaps were raised during the surgical procedure and the authors speculate that the surgery may have disrupted development of these three primary teeth [17].

Histopathologically, CE comprises of large round cells with granular eosinophilic cytoplasm in a fibrous connective tissue stroma. The overlying surface epithelium exhibits atrophy of the rete ridges [4]. There exists much controversy and uncertainty over the histogenesis of CE [18]. The origin of the lesion has been theorized from various tissue components including odontogenic epithelium, mesenchymal cells as well as neurogenic cells [18].

CE is usually an isolated finding and it has not been found to be pathognomic of any other medical condition or syndrome. However, a thorough review of case reports of CE revealed CE occurring in infants with polydactyly [10], goiter [19], Triple X syndrome [10], polyhydraminos [20,21], maxillary hypoplasia [21] and neurofibromatosis [22].

Recent advances in pre-natal imaging have enabled assessment of fetal swallowing and airway patency with the use of 3D ultrasound [8,23]. This enables the physician to plan for a multi-disciplinary team to be present at the time of delivery of the child. This team usually comprises of the obstetrician, obstetric anesthesiologist, pediatric anesthesiologist, neonatologist, otolaryngologist, neonatal nurse and pathologist [5,8]. The option of *ex utero *intra-partum treatment may be planned in cases with obstructive lesions diagnosed in the fetus in the pre-natal period [8,23]. Follow-up of fetuses with CE diagnosed in the pre-natal period with 3D ultrasound has also revealed that this lesion exhibits maximum growth in the end of the third trimester; it has been speculated that hormonal influences towards the end of pregnancy influence this rapid growth of the lesion [10].

Of the more than 200 cases of CE of the new born reported in the English literature, there have been eight case reports [14-16,24-27] that have documented spontaneous regression (Table 1). There have been recommendations in the literature to assume an expectant, non-surgical approach in cases of CE where there is no interference with feeding or respiration [15]. In such cases, regular monitoring of the lesion for regression has been advocated as an acceptable clinical approach [15,16,25]. The reasoning is that the CE has an inherent tendency to involute without exhibiting post-natal growth [12]. In the management of an infant with this lesion, the risks arising from the use of general anesthesia must be weighed in making a treatment decision.

**Table 1:** Reports of cases of congenital epulis managed conservatively

Author	Patient gender	Lesion size	Lesion site	Management	Follow-up duration	Outcome
O'Brien & Pielou 1971[24]	Case 1: male	NS	Maxillary right alveolar process	1 surgically excised, 1 not excised	13 months	Non-resected lesion resolved, dentition unaffected
	
	Case 2: female	NS	Mandibular left posterior alveolar process	1 surgically excised, 1 not excised	12 months	Non-resected lesion disappeared

Welbury 1980 [15]	Female	1 cm	Mandibular right anterior alveolar process	Nonsurgical management	5 years	Residual swelling; dentition unaffected

Jenkins 1989 [25]	Female	1.5 cm	Right maxillary alveolar process	Nonsurgical management	12 months	Lesion size 3-4 mm; dentition unaffected

Marakoglu 2002 [16]	Female	8 × 4 × 4 mm	Anterior mandibular ridge	Nonsurgical management	NS	NS

Sakai 2007 [26]	Female	1.4 × 1.2 × 1.2 cm	Right maxillary alveolar process	Nonsurgical management	10 months	Lesion regressed in 8 months

Ruschel 2008 [27]	Female	1 × 0.6 cm	Left maxillary aanterior alveolar process	Nonsurgical management	12 months	Complete regression at 12 months; dentition unaffected

Dr Erwin Turner 2009[3]*	NS	NS	Right maxillary alveolar process	Nonsurgical management	1 year	Complete regression, dentition unaffected

Ritwik 2009 (current case report)	Female	1.5 cm	Right maxillary alveolar process	Nonsurgical management	16 months	Residual 2 mm swelling, dentition unaffected

Our patient presented with a relatively small lesion (1.5 cm in its greatest dimension) on the maxillary alveolar process, which was not causing any problems with feeding or respiration. With radiographic and urinary investigations we ruled out neuroectodermal tumor of infancy which also has a maxillary anterior site predilection [3]. Parental compliance was excellent for all follow-up appointments. This case demonstrates the ability of the CE of the new born to spontaneously regress. The biologic behavior of the lesion in our patient can be compared to that reported by Welbury [15] and Jenkins [25]. In their case reports, the CE lesions regressed over a period of 12 months, but a residual lesion persisted in the original site. The size of the lesion in our patient was comparable to that reported in all other eight cases of CE which were managed conservatively [4,15,16,24-27]

## Conclusions

It may be concluded that if a CE lesion is less than 2 cm in its largest dimensions and the lesion does not interfere with respiration or feeding, non-surgical management of the lesions ought to be considered. The advantage of conservative management of such cases is to avoid exposure of general anesthesia in a neonate for a lesion which is known to be benign and will not recur. Clinical judgment should be exercised in deciding which cases of CE to monitor for regression and which ones to consider for surgical excision.

## Abbreviations

CE: congenital epulis.

## Competing interests

The authors declare that they have no competing interests.

## Consent

Written informed consent was obtained from the mother of the patient for publication of this case report and any accompanying images. A copy of the written consent is available for review by the Editor-in-Chief of this journal.

## Authors' contributions

PR examined and treated the patient. RBB provided oral and maxillofacial pathology consultation. RJM provided pediatric dentistry consultation. All authors have read and approved the final manuscript.
